# TNBC: Potential Targeting of Multiple Receptors for a Therapeutic Breakthrough, Nanomedicine, and Immunotherapy

**DOI:** 10.3390/biomedicines9080876

**Published:** 2021-07-23

**Authors:** Desh Deepak Singh, Dharmendra Kumar Yadav

**Affiliations:** 1 Amity Institute of Biotechnology, Amity University Rajasthan, Jaipur 303002, India; ddsbms@gmail.com; 2 Department of Pharmacy and Gachon Institute of Pharmaceutical Science, College of Pharmacy, Gachon University, Hambakmoeiro 191, Yeonsu-gu, Incheon 21924, Korea

**Keywords:** triple-negative breast cancer, therapeutic target, signaling pathway, clinical trial

## Abstract

Triple-negative breast cancer (TNBC) is a heterogeneous, recurring cancer associated with a high rate of metastasis, poor prognosis, and lack of therapeutic targets. Although target-based therapeutic options are approved for other cancers, only limited therapeutic options are available for TNBC. Cell signaling and receptor-specific targets are reportedly effective in patients with TNBC under specific clinical conditions. However, most of these cancers are unresponsive, and there is a requirement for more effective treatment modalities. Further, there is a lack of effective biomarkers that can distinguish TNBC from other BC subtypes. ER, PR, and HER2 help identify TNBC and are widely used to identify patients who are most likely to respond to diverse therapeutic strategies. In this review, we discuss the possible treatment options for TNBC based on its inherent subtype receptors and pathways, such as p53 signaling, AKT signaling, cell cycle regulation, DNA damage, and programmed cell death, which play essential roles at multiple stages of TNBC development. We focus on poly-ADP ribose polymerase 1, androgen receptor, vascular endothelial growth factor receptor, and epidermal growth factor receptor as well as the application of nanomedicine and immunotherapy in TNBC and discuss their potential applications in drug development for TNBC.

## 1. Introduction

**Figure 1:**
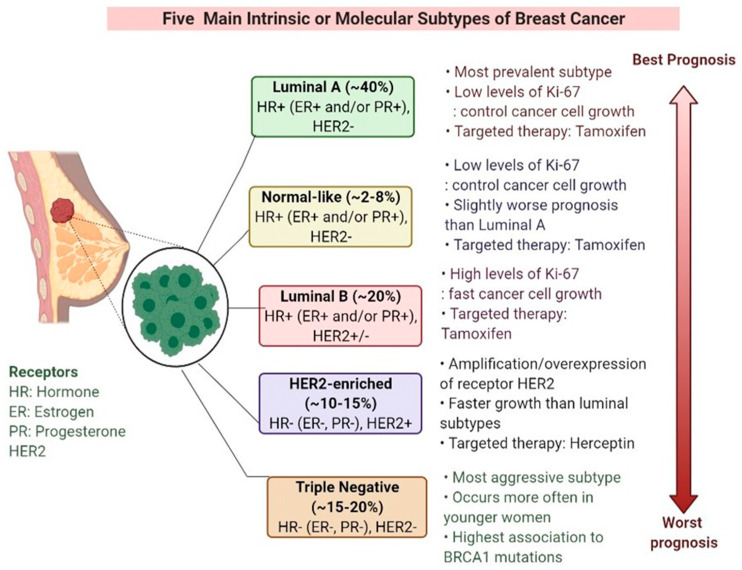
Molecular classification of breast cancer.

**Figure 2:**
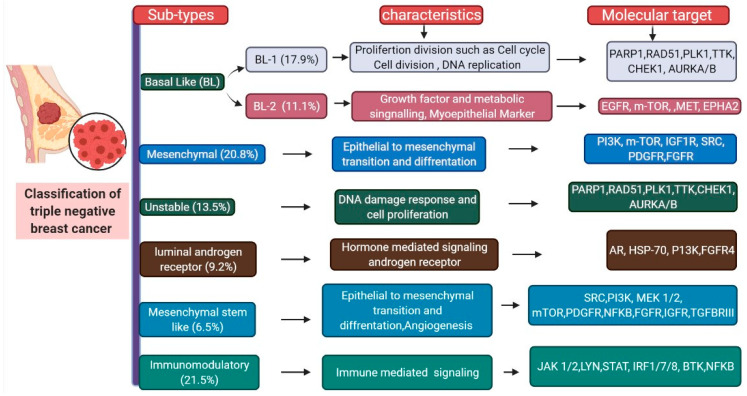
Classification of triple-negative breast cancer.

Breast cancer (BC) is the most common type of cancer in women worldwide. The molecular classification of BC is shown in Figure 1. The highest mortality rate has been observed in triple-negative breast cancer (TNBC). TNBC is characterized by a high histological grade and proliferation rate and ductal histology and is associated with a lack of estrogen receptor (ER), progesterone receptor (PR), and human epidermal growth factor receptor-2 (HER2) expression (making it ER-, PR-, and HER2-negative). The classification of TNBC is shown in Figure 2 [1]. Metastatic TNBC (mTNBC) is associated with a poor overall survival rate [2]. TNBC has a high recurrence rate, which is the greatest within the first 3 years. However, a sharp reduction in recurrence is observed after 5 years. Therefore, there is a lack of long post-therapy regimens [2,3]. Ductal pathology and gene expression analyses have led to further classification of BC into HER2-enhanced, luminal A and B, basal-like, and claudin-low subtypes [1,4]. The claudin-low subtype is primarily diagnosed in women under 45 years of age and is identified by a high expression of epithelial-to-mesenchymal transition-associated genes, low expression of hormone receptor (HR), and low expression of tight junction markers [5]. Currently, TNBC diagnosis is based on mammography, immunohistochemistry, and radio-imaging. Confirmatory biopsy of metastatic lesions is required, as metastatic lesions possess different phenotypes based on the tumor type [6]. Surgery is sometimes recommended to treat TNBC [7].

TNBC has been the subject of intensive research on new therapeutic approaches in recent years [3]. The development of targeted cancer therapies, often in combination with established chemotherapy, has been applied to a few new clinical studies [8,9]. Therefore, there is an urgent need to develop novel therapeutic options. Capecitabine has been used in combination with docetaxel, ixabepilone, doxorubicin cyclophosphamide, and paclitaxel in metastatic TNBC [1,2,3,4,5,6,7,8]. Many studies have been performed to determine whether patients with TNBC were more likely to choose mastectomy over lumpectomy [7]. Results revealed that the TN status, while being associated with younger age and higher-grade tumors, did not impact the patients’ choice of surgical treatment [5,6,7]. Although TNBC tends to be more aggressive, decision-making for surgery likely rests on more traditional clinicopathological variables and patient preferences in disputative TNBC [6]. Traditionally, radiotherapy is administered in TNBC, as in other breast cancer subtypes, following mastectomy or conservative breast surgery (CBS); however, this issue remains controversial [10].

**Figure 3:**
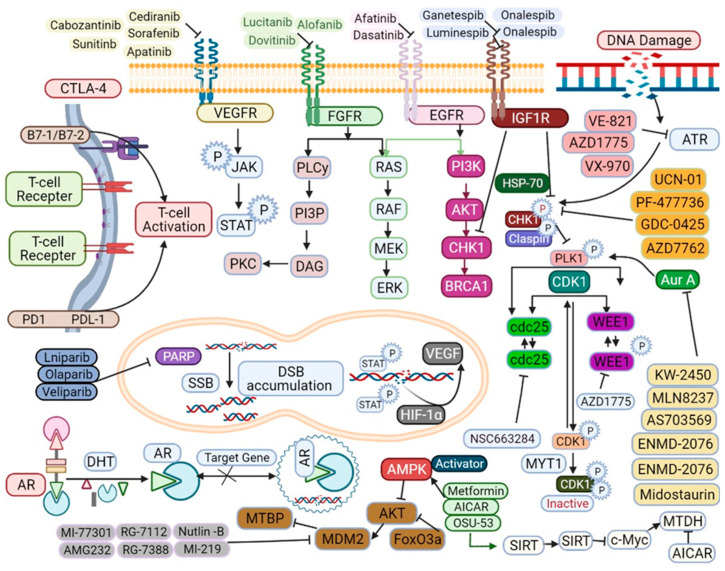
Representation of TNBC-associated molecular targets and their small molecule inhibitors. The arrows represent excitatory regulation, Reversible arrows represent reversible effect of regulation and the headed line arrows represent inhibitory effects.

As TNBC is a rapidly growing and locally aggressive cancer, CBS followed by radiation therapy in early stage (T_1–2_N_0_) may not be equivalent to mastectomy as in other types of BC [10]. Based on the results of metastatic lesion biopsies, TNBC-associated molecular targets and their small molecule inhibitors are shown in Figure 3. In addition to the intrinsic evolutional drive in TNBC, anticancer treatment serves as a source of selection pressure [10,11]. To this date, it remains controversial whether chemotherapy resistance emerges from the selection and expansion of rare pre-existing subclones (adaptive resistance) or from the induction of new mutations (acquired resistance) [11]. Currently, the treatment options for TNBC are limited, because TNBC tumors are not sensitive to hormone therapy and TNBC-specific drug targets are lacking. Some common chemotherapeutic agents show limited efficacy [2,6,10,11]. Therefore, the development of therapeutic options for TNBC is urgently required. In this review, we discuss the diverse TNBC subtypes and examine therapeutic strategies for these subtypes by focusing on platinum-based therapy and the potential of poly-ADP ribose polymerase 1 (PARP1), androgen receptor (AR), vascular endothelial growth factor receptor (VEGFR), and epidermal growth factor receptor (EGFR) under specific clinical conditions, as shown in Figure 3.

## 2. Platinum-Based Chemotherapy

BL1 anchorages a deficiency in HR (homologous recombination) repair, which is mainly driven by mutations or epigenetic changes in the BRCA1/2. The BL2 subgroup, on the other hand, is exclusively improved in development issue signaling pathways such as EGF, NGF, and MET pathways [12,13,14,15,16]. Consequently, directing DNA repair deficit by DNA damage mediators looks to be a gifted action for BL-TNBC. Satisfactory response rates to platinum-based chemotherapy have been related to low BRCA1 mRNA levels and high BRCA1 methylation [12,13,14,15,16]. Platinum-based chemotherapy has been reported to increase the pathological complete response (pCR) rate in TNBC patients [15]. A phase III randomized clinical trial was conducted; the treatment included six cycles of paclitaxel plus carboplatin (PCB) with a standard-dose regimen of three cycles of cyclophosphamide, epirubicin, and fluorouracil followed by three cycles of docetaxel (CEF-T) [NCT04127019]. A total of 647 patients (mean (SD) age, 51 (15) years) with operable TNBC were randomized to receive CEF-T (*n* = 322) or PCB (*n* = 325). At a median follow-up period of 62 months, the DFS was longer in patients administered PCB than in patients administered CEF-T (5-year DFS, 86.5% vs. 80.3%, hazard ratio (HR) = 0.65; 95% CI, 0.44–0.96; *p* = 0.03) [15,16]. Safety data were consistent with the known safety profiles of relevant drugs. The primary endpoint was disease-free survival (DFS). Secondary endpoints included overall survival, distant DFS, relapse-free survival, DFS in patients with germline variants in *BRCA1/2* or homologous recombination repair (HRR)-related genes, and toxicity [14,16]. Platinum salts have been increasingly tested for TNBC in combination with various other chemotherapy drugs (e.g., gemcitabine, which masquerades as cytidine and inhibits DNA synthesis) [12]. Moreover, identifying predictive biomarkers is imperative for the selection of appropriate patients for platinum-based regimens in the adjuvant setting.

## 3. Targets of TNBC under Active Clinical Evaluation

### 3.1. Poly (ADP Ribose) Polymerase Inhibitors

**Figure 4:**
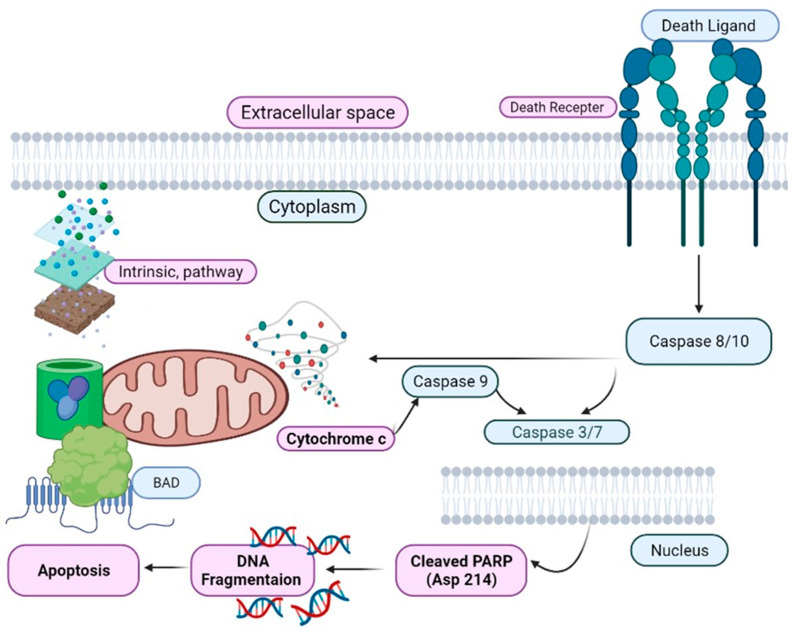
Expression of PARP via Caspase predicts good chemotherapy response and poor survival for patients with TNBC.

PARP inhibitors are actively involved in HR-repair deficiency and responding to ss- DNA damage and continue genomic integrity by using BER (Base Excision Repair Mechanism) [12,13,17]. Ds-DNA damage is typically repaired via HR, which requires normal functions of the tumor suppressor proteins BRCA1/2 [17]. Thus, the use of PARP inhibitors shows promise in the treatment of TNBC with HR deficiency; this approach does not result in side effects on remaining normal cells [18]. Olaparib (a PARP inhibitor) has been reported to prevent the development of BRCA-related metastatic tumors [18]. PARP plays an important role in maintaining the genome stability, chromosome number, DNA repair process, and cell cycle and transcription regulation, as shown in Figure 4 [19]. PARP inhibitors are novel targeted anticancer drugs, and many clinical studies on PARP inhibitors have been accomplished. Various agents, such as olaparib (AZD2281, AstraZeneca/KuDOS) and BSI-201 (BiPAR Sciences/Sanofi Aventis), are currently in the initial stage of clinical trials, as shown in Table 1.

**Figure 5:**
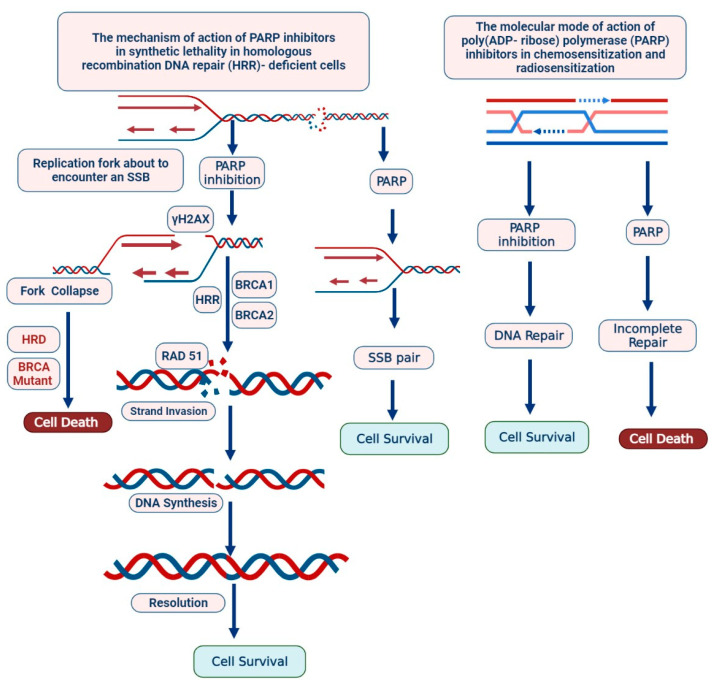
The mechanism of PARP inhibitors is synthetic lethality in homologous recombination DNA repair (HRR) deficient cells. The molecular mode of action of poly (ADP ribose) polymerase inhibitors in chemosensitization and radiosensitization.

The PARPi response is determined by the genetic status of a patient; *APEX1*, *PCNA*, *PCLB*, *RPC1*, *RPC3*, *RPC4*, *RPA1*, and *FEN1* have been linked with PARPi, HR, *BRCA* mutations, and DNA damage response; however, patient-derived xenograft models are required to analyze PARPi sensitivity in TNBC, as shown in Figure 3, Figure 4 and Figure 5. The clinical impact of olaparib, an oral PARPi, has been investigated in phase I trials (NCT04239014) in *BRCA*-mutated patients with advanced tumors [13,14,15]. Pharmacokinetic and pharmacodynamic data confirmed PARP inhibition, and no adverse effects were observed. Additionally, a cohort-type, multicentric, phase II clinical trial (NCT02734004) was performed to determine the efficacy and tolerability of olaparib in patients with *BRCA1*- and/or *BRCA2*-deficient advanced breast cancer [14,15]. The majority of the patients harbored *BRCA1* mutations, and more than 50% presented with TNBC. Olaparib has also been evaluated in phase III BC trials [9,13,14,15].

Rucaparib is an effective inhibitor of PARP1, PARP-2, and PARP-3 in BRCA-mutated patients (germline and/or somatic) [17,18,19,20]. Rucaparib was also found to be effective in HR-deficient patients. Rucaparib is considered in monotherapy treatment of adults with platinum-sensitive tumors, patients who have been treated with two or more prior lines of platinum-based chemotherapy, and patients who are unable to tolerate further platinum-based chemotherapy. The efficacy and safety of rucaparib in patients with HER2-negative metastatic breast cancer were associated with a BRCAness phenotype and/or a somatic BRCA mutation [20]. Patients received 600 mg orally for 21 days or up to the development of disease [18,19]. The primary endpoint was the clinical benefit rate, and the secondary endpoints included PFS, overall survival, safety, and prognostic value of the BRCAness signature. Additional studies were performed to determine the number of sporadic TNBC patients likely to benefit from rucaparib treatment. Rucaparib, a PARPi, was evaluated and approved by the Food and Drug Administration (FDA) in 2016 for patients with germline *BRCA* mutation (gBRCA) [20].

Talazoparib has also been approved for patients with gBRCA mutation [19]. Currently, PARP inhibitors are considered in various combination treatments with cytotoxic agents and radiotherapy. PARP inhibition is studied in patients with the BRCAness phenotype, which could lead to effective clinical management in patients with TNBC. Since 30% of sporadic tumors possess the BRCAness phenotype, clinical trials must investigate whether there is an increased antitumor effect when combining these agents, with manageable side effects. When pharmacodynamic assays are generally applied in treatment with PARP inhibitors, under- and over-dosing could be prevented; however, this concept needs prospective clinical validation.

Developing effective clinical strategies and increasing PARPi sensitivity may help overcome drug resistance. PARPi has been reported to promote radiosensitization in an animal model as well as in cell lines [13,14,15]. Preclinical model systems showed increased radiosensitivity due to HR restoration via 53BP1 pathway inactivation. HR is a complex process, requiring a myriad of proteins [12]. The MRN-complex, composed of MRE11, Rad50, and Nbs1, plays several roles in the DNA damage response. The most well recognized is the role of the MRN-complex, which acts as a sensor of DSBs to initiate HR following their detection [12,13,17]. The MRN-complex is rapidly recruited to the sites of DSBs, facilitating the recruitment and activation of ATM kinase and initiating the subsequent ATM-mediated phosphorylation of each member of the MRN-complex. This promotes further recruitment of the MRN-complex and initiates ATM-dependent downstream signaling [18,19,20].

It was observed that BRCA1-mutated tumors led to drug resistance due to BRCA1-independent HR restoration and sensitization to radiotherapy [10]. PARPi was also used in combination with HSP90 inhibitors, WEE1 inhibitors, and ATR/CHK1 inhibitors. HSP90 plays an important role in BRCA1 function [13]. The HSP90 inhibitor (7-dimethylaminoethylamino-17-demethoxygeldanamycin) reverses the resistance state by decreasing the levels of BRCA1 protein [15]. WEE1 inhibitors and ATR/CHK1 treatment also play an important role in reversing PARPi resistance. [18] BSI was investigated as a monotherapeutic agent and in combination with other DNA-damaging anticancer agents in a phase I clinical trial (NCT03524261). Consequently, PARP activity was found to be suppressed [12,15].

### 3.2. EGFR

EGFR is a transmembrane receptor that stimulates growth factor signaling pathways as shown in Figure 3. EGFR receptors, such as HER1, HER2, HER3, and HER4, actively participate in cell cycle regulation, differentiation, proliferation, and survival [19]. TNBC tumors are widely assessed as basal-like tumors, because of the overexpression of *EGFR* and reduced expression of *BRCA1* and miR-146a. *EGFR*-targeted therapies are based on tyrosine kinase inhibitors (TKIs), monoclonal antibodies, and combination chemotherapy [20]. However, ongoing clinical trials for the same have revealed limited responses, and many EGFR inhibitors are currently undergoing clinical trials. Combined therapy with afatinib and dasatinib has been used to inhibit both ERK and Akt signaling. Several patients with TNBC do not respond to metastatic disease. [20,21]. Dasatinib is an Src family kinase inhibitor that prevents cell cycle progression, proliferation, and translocation of EGFR [21]. Cetuximab and ixabepilone are microtubule-targeting drugs that are effective in patients with mTNBC [20]. Cetuximab inhibits the growth of TNBC tumors by blocking the ligand-induced phosphorylation of EGFR. Lapatinib and gefitinib are EGFR-TKIs that have also shown anti-proliferative activity in studies [20,22,23]. Investigations of PI3K, MEK1/2, Akt, and small interfering RNA are also molecules/pathways as shown in Figure 3. Monoclonal antibodies have shown EGFR antitumor activity by inhibiting cell signaling pathways, dimerization, and ligand receptors [15]. Neratinib, an irreversible pan-HER inhibitor, has shown effective responses in clinical trials (NCT01953926) [15,20]. The targeting of MET, a regulator of EGFR tyrosine kinase phosphorylation, has been combined with fulvestrant in HR-positive BC [19,20]. Erlotinib, a TKI, in combination with rapamycin can reduce tumor growth [3,16]. High expression of the RAS/MEK/ERK pathway has been observed in patients with TNBC, and this signaling pathway may be an effective therapeutic target for TNBC as well [24,25]. Selumetinib and gefitinib can inhibit cell cycle arrest and apoptosis and have shown significant results in TNBC cell lines [24,25,26]. Further, the tumor microenvironment has been investigated as a novel therapeutic target. Thus, further investigations are required to understand EGFR-based targeted therapies and the adaptive immune system in patients with TNBC as shown in Figure 3.

### 3.3. Fibroblast Growth Factor (FGF)

The fibroblast growth factor receptor (FGFR) signaling cascade plays a pivotal role in cell proliferation, differentiation, apoptosis, and migration [27,28]. FGF ligands bind to FGFRs, leading to the dimerization and regulation of the PI3K/AKT, MAPK, STAT, IP3-Ca^2^+, and DAG-PKC pathways as shown in Figure 3. Despite widespread preclinical analysis on all main RTKs (Figure 3), limited studies have emphasized the possible benefits of directing c-MET, AXL, and the EGFR family of RTKs in treating TNBC patients [29]. c-MET and AXL cooperate physically in TNBC cells, AXL suggestively expands EGFR signaling and limits the response to EGFR-targeted inhibitors in TNBC cells [30]. AXL systems form a complex with additional HER family members, as well as with c-MET and PDGFR in TNBC cells, further suggesting a widespread role of these RTKs in TNBCs [31,32]. FGFR2 is amplified in TNBC, and interference with FGF signaling using FGFR inhibition has been shown to significantly impair tumor formation in xenografts, further suggesting that it may be a viable target for the treatment of a subset of TNBCs [32,33]. Lucitanib has been investigated for its effect against *FGFR1* amplification in xenograft models, and phase II clinical trials (NCT02109016) have shown a significant objective response rate (ORR) in patients with HER2-negative, HR-positive, and high *FGFR1* expression [34]. Clinical trials on rucaparib, a VEGF and PARP inhibitor, are underway. The clinical potential of NVP-BGJ398 and AZD4547, which are FGFR inhibitors, is under phase I clinical evaluation (NCT01004224) [35]. AZD4547 has shown limited efficacy against *FGFR1*, *FGFR2*, and *FGFR3*, and low efficacy against *FGFR4* [36]. In phase II clinical trial (NCT01202591), the efficacy and toxicity of fulvestrant were evaluated in ER-positive patients. In a phase III clinical trial (NCT01795768), patients with esophageal cancer, lung carcinoma, and gastric cancer overexpressing *FGFR1* or *FGFR2* were recruited [37]. The safety level was measured by evaluating ERK phosphorylation [37]. Dovitinib (TKI258) is an inhibitor of multiple kinases, including FGFR, VEGFR, and platelet-derived growth factor receptor (PDGFR); its efficacy has been proven in HER2-negative metastatic breast cancer (NCT00958971), and it inhibits the invasion of MDA-MB-231(SA) cells [38,39]. E-3810 inhibits *FGFR1*, *FGFR2*, *CSF1R*, *VEGFR1*, *VEGFR2*, and *VEGFR3I* [40]. Ponatinib inhibits BCR-ABL and its activity has been evaluated in BC cell lines in vitro [41]. AP24534 inhibits the phosphorylation of FGFR; however, further clinical evaluation is required to determine its efficacy. GP369 is a potent inhibitor of FGFR2 in cancer cells in vitro [42]. Lenvatinib is a potential molecular target of KIT, PDGFR-α, and FGFR, and it shows antitumor activity in HCC (hepatocellular carcinoma) by targeting FGF/FGFR signaling; however, further investigation into its anti-FGFR activity is required [43]. Infigratinib is a pan-FGFR inhibitor, a phase II clinical trial (NCT03773302) has shown significant results, efficacy was compared with gemcitabine and cisplatin in a patient with TNBC (*FGFR2* gene fusions and translocations) [44]. Thus, further development and evaluation of FGFR inhibitors using combination therapy can be an effective targeted therapeutic strategy for patients with TNBC.

### 3.4. AR

AR is expressed in TNBC tumors and plays a role in suppressing apoptosis and cell proliferation as shown in Figure 3 [45]. AR is activated by signal transduction in an ERK-dependent (ERKD) or independent manner. In ERKD AR signaling, cytoplasmic AR interacts with Src proteins, Ras GTPase, and phosphoinositide 3-kinase (PI3K) TNBC as shown in Figure 3 [46]. AR-supplemented TNBC cell lines commonly transmit PI3KCA mutations, which make them very effective for PI3K/mTOR inhibition. AR mutations in the kinase domain increase *PTEN* expression [46,47,48,49]. Increased *PTEN* expression regulates the expression of protein killin (KLLN) and promotes p53 and p73 expression, subsequently augmenting apoptosis [50,51]. GATA-3, an important transcription factor, is involved in luminal cell differentiation and restricts the effects of drugs by enhancing ER signaling activity [49]. Further, GATA-3 expression has been closely associated with apocrine TNBC [49]. The antagonist bicalutamide was the first AR-based drug that was clinically evaluated in 2013; however, limited efficacy and adverse effects, such as limb edema, fatigue, and hot flashes, were observed. Enzalutamide has been clinically evaluated in patients with AR-positive TNBC, and the most common adverse effects observed were fatigue and nausea [52]. The PFS and safety of abiraterone, a selective inhibitor of CYP17 was clinically evaluated, and hypokalemia and hypertension were the most common adverse events. A clinical trial (NCT01889238) of bicalutamide and palbociclib showed effective clinical data for their administration alone and in combination with other drugs in patients with TNBC [53]. Seviteronel is a CYP17-L inhibitor and is in phase II clinical development (NCT02580448) for TNBC treatment [54].

### 3.5. PDGF/VEGFR

The PDGF (platelet-derived growth factor) family is composed of four members, PDGF-A, PDGF-B, PDGF-C, and PDGF-D, which bind either as homo- or heterodimers to one of the two RTKs, PDGFR-/or PDGFR-b, to regulate cell migration, proliferation, and survival [55]. Overexpression of PDGF and VEGF is highly expressed in TNBC [55,56]. PDGF signaling induces self-renewal capacity in differentiated cancer cells, enabling them to behave like cancer stem cells via PKC/-dependent activation of FOS-like antigen 1 (FRA1) [56]. Imatinib is approved by the US FDA for the treatment of chronic myeloid leukemia (CML) and targets the phosphorylation of RTKs including PDGFR-b and v-Kit Hardy–Zuckerman 4 feline sarcoma viral oncogene homolog (c-KIT) [57]. Both the monoclonal antibody bevacizumab, which specifically targets VEGF, VEGFR is a major factor responsible for vasculogenesis and angiogenesis as shown in Figure 3 [58]. VEGF can induce immunosuppression by inhibiting the development of cytotoxic T lymphocytes and dendritic cells and increasing the recruitment and proliferation of immunosuppressive cells [59]. Sixty percent of TNBC cases show high *VEGF-A* expression, and mesenchymal stem-like TNBC tumors show high *VEGF-C* expression; in such cases, survival is poor [60]. Bevacizumab and ramucirumab block the activation of VEGF TNBC as shown in Figure 3 [61]. In a phase III clinical trial (NCT01004172) in patients with TNBC and metastatic tumors, bevacizumab was investigated in combination with epirubicin, cyclophosphamide, and docetaxel. Although more substantial results were observed against HER2-negative metastatic tumors using combination therapy than monotherapy, the results were not statistically significant [62]. Another clinical trial of bevacizumab in combination with a taxane, gemcitabine, capecitabine, or vinorelbine revealed enhanced ORR [61,62]. Bevacizumab was also clinically evaluated in combination with nab-paclitaxel, carboplatin, and bevacizumab in patients with mTNBC [61,62]. However, the clinical outcomes of the above-mentioned trials (NCT00861705, NCT00608972, NCT02456857, NCT01094184, and NCT00472693) have not been reported [63]. Aflibercept is a tyrosine kinase that acts on receptor tyrosine kinases. Ramucirumab in combination with docetaxel is also undergoing a clinical trial. Temsirolimus, an mTOR inhibitor, has shown significant improvements in ORR [64]. Sorafenib is a VEGFR TKI that induces a significant improvement in patients with TNBC; however, it does not show efficacy in combination with bevacizumab (BRE06-109) [65]. The efficacy of cediranib (AZD2171) with olaparib has been tested in a phase I trial; however, no significant clinical benefits have been observed. Apatinib has been clinically examined for the treatment of mTNBC [66]. Cabozantinib (XL184) inhibited the growth and invasion of TNBC in preclinical models as a monotherapeutic agent with limited clinical benefits [67]. Sunitinib is an inhibitor of PDGFR, c-Kit, and colony-stimulating factor 1 receptor; TNBC progression has been observed after withdrawal of sunitinib. Interestingly, considerable progress has been made in understanding the regulation of VEGFR-2 expression [68]. Clinical evaluation of the above-mentioned drugs in combination or alone can be explored as an effective therapeutic strategy for TNBC as shown in Figure 3.

### 3.6. Other Promising Therapeutic Targets

Targeting various pathways has become a major focus for an anticancer chemotherapeutic agent such as DNA damage-induced cell cycle arrest, DNA damage checkpoint kinases including CHK1/2 (checkpoint kinase 1/2), ATR (ataxia telangiectasia and rad3-related protein), and ATM (ataxia telangiectasia mutated) [69,70,71,72,73].

#### 3.6.1. Inhibition of CHK1/2

CHK1 is essential for checkpoint-mediated cell cycle capture in reply to DNA damage or the presence of unreplicated DNA [70]. CHK1 is overexpressed in rapidly dividing and gnomically unstable cells, as is predictable in TNBC cells. Based on the genomic and clinical trial data analysis that CHK1 is a draggability target, other CHK1 inhibitors AZD7762 (NCT00937664), PF-477736 (NCT03057145), SCH900776 (NCT00907517), and LY2606368 (NCT02203513) are currently under clinical trials. CHK2 inhibitor LY2606368 (NCT02124148) together with chemotherapy is currently under trial in patients with TNBC [70].

#### 3.6.2. Inhibition of CDKs

Cyclin-dependent kinases (CDKs) are triggered via cyclins that allow progress through the cell cycle. CDKs are repressed by logically happening CDK inhibitors, but in carcinogenesis, CDK inhibitors are overexpressed and lead to uncontrolled cell proliferation [72]. Numerous CDK inhibitors have been developed and directly inhibit CDK2, CDK4, and CDK6, and inhibit apoptosis. CYC202 has been shown to have in vivo activity against CDK1 and CDK2 in TNBC [30]. CDK4/6 has shown inhibition in PIK3CA-mutant xenograft tumor models and CDK4/6 inhibition has shown growth retardation. CDK activity is required for resection of DSBs (double-stranded break) and to repair damage by HR (homologous recombination) [30,72]. The inhibition of CDK1 sensitizes for extending the utility of PARP inhibitors to BRCA1/2-proficient cells.

#### 3.6.3. PI3K Inhibitors

The PI3K/AKT signaling pathway is frequently hyperactivated in TNBC due to PIK3CA or AKT1 mutations and/or PTEN inactivation [73]. AKT inhibition is increased chemosensitivity in TNBC, eventually overcoming chemoresistance in this disease subset. Hence, several trials have investigated AKT inhibitors in association with chemotherapy for TNBC [73]. Two randomized placebo-controlled phases II trials evaluated the combination of an ATP-competitive inhibitor such as ipatasertib and capivasertib with weekly paclitaxel for the first-line treatment of advanced TNBC [74]. The PAKT trial randomized 140 patients to receive capivasertib plus paclitaxel (*n* = 70) or placebo plus paclitaxel (*n* = 70). The primary endpoint was median PFS in the intention-to-treat population and it was numerically longer in the experimental arm (5.9 months) compared to the control arm (4.2 months) (HR 0.74; 95% CI: 0.5–1.08, one-sided *p* = 0.06) [75]. However, progression-free survival was significantly extended with capivasertib in the PIK3CA/AKT1/PTEN mutated subpopulation (9.3 months vs. 3.7 months; HR 0.3; 95% CI: 0.11–0.79; p 0.1). Updated results after 40 months of follow-up showed a favorable trend in terms of OS for capivasertib plus paclitaxel, regardless of the PIK3CA/AKT/PTEN mutational status (median OS in the overall population 19.1 months vs. 13.5 months; HR 0.7; 95% CI: 0.47–1.05; *p* = 0.085) [74]. Additionally, the combination of ipatasertib with a non-taxane-based chemotherapy in mTNBC patients is currently under evaluation in the phase II PATHFINDER trial [75,76]. In the early-stage setting, a phase II randomized trial evaluated the use of AKT inhibitors in TNBC.

## 4. Nanomedicines for TNBC

Nanotechnology can be used to develop nanoparticles (NPs) with functional properties for therapeutic applications [77,78]. These functional properties typically include surface charge, particle size, and conformation for specific targeted drug delivery using a receptor-specific target in a cancerous cell [78]. Functionalized NPs are fabricated from various materials, such as gold, silver, diamond, and copper (Table 2). Antibodies (anti-EGFR and anti-VEGFR) are considered the best class of targeting ligands [61]. Antibodies conjugated with fluorescent NPs and radio-imaging contrast agents can be detected using fluorescence microscopy and ultrasonography [79]. A preclinical study on TNBC xenograft mice demonstrated good visualization of TNBC tumor virus-like particles produced by the expression of viral structural genes [80]. Therefore, Nanomedine may provide hope for TNBC treatment by improving on classical chemotherapy. A preclinical study in animal models with TNBC demonstrated that labeled antibodies show a good treatment response [80,81]. Various nanomedicines for TNBC theranostics are shown in Table 3. Liposome-based NPs carrying doxorubicin and rapamycin with cyclic octapeptide LYX (Cys-Asp-Gly-Phe (3,5-DiF)-Gly-Hyp-Asn-Cys) have been investigated. Irinotecan (SN-38) with NK012 (NCT00951054) micelle is currently undergoing a phase II clinical trial in patients with TNBC (Table 3) [64]. siRNA-conjugated poly(amidoamine) dendrimers have shown the downregulation of the TWIST transcription factor in patients with TNBC [81,82]. The Gd-DOTA (42-G4 PAMAM-DL680) dendrimeric agent has been inserted hypodermically into mice for imaging and drug delivery purposes. L-lactic-co-glycolic-acid, a polymeric nanoparticle, shows a high degree of tumor inhibition in vivo in TNBC mouse models [83]. Poloxamer (P188) with succinobucol inhibits vascular cell adhesion molecule-1 invasion and cell migration. RGD-SLN or RGD-functionalized solid lipid NPs have shown efficacy in a TNBC animal model [77,83,84]. Differential overexpression of platelet-derived growth factor (PDGF) receptor in the TNBC cell line was detected by using conjugated gold NPs [85]. Many clinical trials of drugs with functionalized NPs are ongoing and are summarized in Table 3 [86]. However, such functionalized targeted therapy and diagnosis still need to be improved and combined with drug delivery for effective TNBC therapeutic applications.

## 5. Immunotherapy

**Figure 6:**
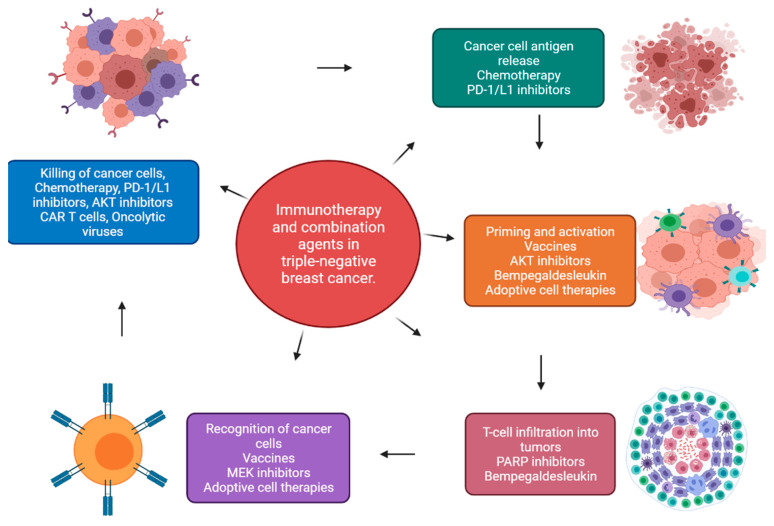
Immunotherapy agents in triple-negative breast cancer.

Immunotherapy for TNBC has accelerated the research on immuno-oncology drugs as shown in Figure 6 [92,93]. The FDA has approved the combination of Tecentriq (atezolizumab) and Abraxane (nab-paclitaxel) for the front-line treatment of patients with metastatic PD-L1-positive TNBC as shown in Figure 3 and Table 4 [92,93]. Notably, the 2-year overall survival (OS) rates were higher in the PD-L1-expressing population at 51% in the atezolizumab arm versus 37% in the as monotherapy (Table 5 and Table 6) and combination therapy (Table 4, Table 5 and Table 6) [94,95,96,97,98]. Further, the combination of AKT and PARPi has been approved for use as a potential immunotherapy pipeline for cancer.

The combination of atezolizumab with nab-paclitaxel has been approved as front-line therapy for patients with metastatic PD-L1-positive TNBC who have received at least 1 year of either adjuvant or neoadjuvant taxane; many patients in this trial presented with de novo metastatic diseases [97]. Impassion130 the trial showed a significant, though modest, improvement in the PFS, but a marked difference in the OS [98]. The tumor microenvironment plays a role in immune cell functions and downregulates antitumor immune responses. For instance, PD-1 and PD-2 are expressed in cytotoxic CD8+ T cells and promote cellular immunity against cancer cells as shown in Table 4 [99]. A phase I clinical trial (NCT04157985) of pembrolizumab and avelumab, which are anti-PD-1 antibodies, revealed partial ORR (objective response rate) in mTNBC (Table 4). High expression of EGFR and PD-L1 is a common phenomenon in TNBC [100,101,102]. In immune cells (IC), PD-L1 is expressed in CD11b+ myeloid cells such as dendritic cells and macrophages mainly but also T cells and NK cells [94,103]. In the Impassion 130 investigating atezolizumab in combination with nab-paclitaxel for mTNBC, PD-L1 IC expression was a stratification parameter [97,98]. The subgroup of patients with PD-L1 > 1% (185/451 patients) benefited particularly from atezolizumab, a trend toward a higher ORR was seen in patients with PD-L1 IC+ vs. patients with PD-L1 IC− in the overall population (16.7% vs. 1.6%) [104]. Nevertheless, the FDA recently granted accelerated approval to atezolizumab in combination with nab-paclitaxel patients with unresectable locally advanced or metastatic TNBC whose tumors express PD-L1 (PD-L1 IC ≥ 1% of the tumor area) [104]. A recent retrospective study examined PD-1 mRNA expression in 10,078 tumor samples representing 34 different cancer types from TCGA and found a significant correlation between PD-1 mRNA and the ORR following anti-PD-1 monotherapy, while PD-L1 tumor expression by IHC or the percentage of TILs were not found to be associated with the response [104]. However, contrary to other tumor types like melanoma and lung cancer, in which recent studies support TMB as a predictive biomarker for ICI efficacy, TMB was not demonstrated as a predictor of ICI efficacy in BC, notably in the Impassion 130 study, but few data are available about TMB and response to immunotherapy in BC [105]. The ORR was 21%, and the disease control rate was 37%, suggesting a certain level of activity of pembrolizumab in this subset of patients [100,101,102]. Moreover, early changes in circulating tumor DNA levels may be associated with a response to ICI. Of note, no tumor-associated antigens (TAAs) have been shown to be associated with the ICI response [102]. More specifically, a translational analysis using single-cell RNA-seq revealed that a specific subset of T cells (CD8+, resident memory) was significantly associated with improved patient survival in early-stage TNBC [105]. Thus, for patients with PD-L1-positive disease, this is an important therapeutic development.

MSI (microsatellite instability) is caused by dMMR (defective DNA mismatch repair) genes and is categorized by an altered in repeated nucleotide sequences, which may enhance to evasion of apoptosis, expansion of mutations, and tumorigenesis [106]. MSI is a marker of dMMR. dMMR and MSI-H have been found in various tumors, such as uterine, central nervous system, and adrenal gland tumors. Both dMMR and high-frequency MSI (MSI-H) have been demonstrated as effective predictors of immunotherapy response, dMMR/MSI-H has been associated with poor prognosis in individuals with colorectal cancer who were insensitive to 5-fluorouracil (FU)-based adjuvant chemotherapy. However, data on the prevalence and the prognostic significance of dMMR/MSI-H in BC is limited, especially for TNBC. Although there have been studies on MMR/MSI status in breast cancer, the number of cases is often small, with the largest cohort comprising 444 patients, only 23 of which were TNBC. The proportion of MSI-H in these groups varied largely (from 0.2% to 18.6%) [106]. Therefore, further verification of the relationship between MMR/MSI status and prognosis is needed.

Immuno-oncology (IO) is a novel approach to cancer treatment by the stimulation of the body’s own immune system [107]. Immune checkpoint inhibitors (ICPis) have had notable achievement across multiple malignancies, and are the most well-established IO agents to date, with several approvals [107,108]. Biomarker testing for the programmed death-ligand 1 checkpoint target is mandatory earlier in treating some tumor types with ICPis (e.g., pembrolizumab and atezolizumab). Combining IO agents with conventional therapies has provided significant improvements in patient outcomes in some cases [109]. The two main challenges for IO agents are managing their toxicities and affording the high cost of these novel therapies. In a recent study, pembrolizumab was administered in a neoadjuvant setting, along with standard chemotherapy comprising paclitaxel and carboplatin followed by doxorubicin, epirubicin, or cyclophosphamide [61]. Currently, trials are investigating combination treatments with an AKT inhibitor [68,74]. One single-arm study aims at randomizing patients to receive ipatasertib and a taxane with or without atezolizumab (Table 5, Table 6 and Table 7) [94,97,98]. Other trials are examining AKT inhibitors, ipatasertib and capivasertib, in combination with checkpoint inhibitors in patients with TNBC who have alterations in the PI3K pathway [73]. The combination of immunotherapy with PARPi is also of great interest [74]. The phase II/III MK-7339-009/KEYLYNK-009 trial is based on randomized patients receiving gemcitabine/carboplatin and pembrolizumab, followed by continuous chemotherapy and maintenance with pembrolizumab or pembrolizumab and olaparib (Lynparza) [88,89,90,91,92,93,94,95,96,97,98,99,100,101].

In the OlympiAD study, olaparib has been shown to improve progression-free survival compared with chemotherapy treatment of physician’s choice (TPC) in patients with a germline BRCA1 and/or BRCA2 mutation (BRCAm) and HER2-negative mBC (metastatic breast cancer) [110]. In the phase III OlympiAD study in patients with a germline BRCA mutation and HER2-negative metastatic BC, a total of 205 patients were randomized to olaparib and 97 to TPC. HR for OS with olaparib versus TPC in prespecified subgroups were prior chemotherapy for mBC receptor status (triple-negative: 0.93, 0.62–1.43; hormone receptor-positive: 0.86, 0.55–1.36); prior platinum (yes: 0.83, 0.49–1.45; no: 0.91, 0.64–1.33) [10]. Adverse events during olaparib treatment were generally low grade and manageable by supportive treatment or dose modification. There was a low rate of treatment discontinuation (4.9%), and the risk of developing anemia did not increase with extended olaparib exposure [110]. The trial has enrolled patients with TNBC as monotherapy and combination therapy as shown in Table 5, Table 6 and Table 7. Administering these agents earlier during therapeutic action is also being investigated.

### 5.1. Antibody-Drug Conjugates (ADCs)

Antibody-drug conjugates (ADCs) are immunoconjugate agents engineered to deliver potent small molecules preferentially to cancer cells [111]. This novel approach combines the specificity of a monoclonal antibody (mAb) with the high potency of small molecules and has the potential to improve TNBC [111]. Since ADCs can provide a broader therapeutic window than conventional chemotherapy, combination therapy with other agents is a potentially effective strategy to enhance synergy as well as target tumor heterogeneity [112,113]. For example, the combination of sacituzumab govitecan with PARP inhibition in TNBC models in vitro and in vivo resulted in increased dsDNA breaks and synergistic growth inhibition regardless of the BRCA1/2 status in a preclinical study [113]. Despite advancements in the development and engineering of ADCs, the majority of ADCs utilize payloads from only a few categories of cytotoxic agents: antimitotic agents, microtubule inhibitors, antitumor antibiotics, and DNA-damaging agents [114]. The largest group of ADCs in clinical trials use antimitotic monomethyl auristatin E (MMAE) and MMAF, owing to their high potency, water solubility, and stability under physiological conditions [115]. The second-largest class of payloads of ADCs in clinical trials is microtubule-inhibiting maytansinoids (DM1 and DM4), which have excellent stability and acceptable water solubility [116]. Calicheamicin is a highly potent antibiotic that binds to the minor groove of DNA and creates double-stranded DNA breaks [117]. Camptothecin analogs, such as SN-38 and exatecan mesylate, are potent DNA-damaging agents that exhibit topoisomerase 1-inhibitory activity [116,117]. Recently, polatuzumab vedotin-piiq, a CD79b-directed ADC carrying MMAE by protease-cleavable peptide linker in combination with bendamustine plus rituximab, was approved for relapsed diffuse large B-cell lymphoma [118]. As a result of these efforts to improve the therapeutic index of the drug by maximizing the tolerated dose and minimizing the effective dose, novel ADCs are emerging for treatment of patients with TNBC.

### 5.2. Tumor-Associated Antigens (TAAs)

Targets for tumor vaccines are divided into two types: tumor-associated antigens (TAAs) and tumor-specific antigens (TSAs) [119]. TAAs are self-antigens and are abnormally expressed in tumor cells, as self-antigens. T cells that bind with a high affinity to TAAs are typically deleted from the immune repertoire via central and peripheral tolerance mechanisms, and thus a cancer vaccine using these antigens must be potent enough to break the immunological tolerance [120]. High-affinity T cells may be present and strongly activated by these antigens. Similarly, neoantigens encoded by genes carrying oncogenic driver mutations may be prevalent across patients and tumor types and hence are referred to as shared neoantigens [121]. The majority of neoantigens are unique to tumors of individual patients (private neoantigens), thus necessitating personalized therapy [120].

### 5.3. Adoptive T-Cell Therapy

Adoptive T-cell therapy, involving the autologous or allogeneic transplantation of tumor-infiltrating lymphocytes or genetically modified T cells expressing novel T-cell receptors or chimeric antigen receptors, has shown promise in the treatment of cancer [122]. Tumor-infiltrating lymphocyte (TIL) therapies are a form of adoptive cell transfer (ACT) immunotherapy in which T cells are grown and expanded from resected metastatic tumor deposits [123,124]. In chimeric antigen receptor (CAR) T-cell therapy, T cells isolated from a patient with cancer are engineered to express tumor antigen-specific receptors that facilitate the elimination of tumor cells upon reintroduction [125]. Two CAR T-cell therapies, tisagenlecleucel (Kymriah) and axicabtagene ciloleucel (Yescarta), have been FDA-approved for hematological malignancies [126]. Rosenberg and others reported successful outcomes of ACT-TIL therapy in metastatic melanoma; these are thought to be partly due to the high acquired/somatic mutational load and the highly immunogenic nature of this cancer [122,123,124,125]. Improvements in high-throughput genetic sequencing have enabled the identification of TIL-targetable mutations in BC [124]. The impressive efficacy of ACT-TIL therapy in metastatic melanoma is highlighted not only by higher ORRs (approximately 50%) but also durable and complete response (CR) rates (13%), which exceed those of some immunotherapies, such as checkpoint-blockade agents in TNBC and HER2-positive BC [123,124,125,126]. T-cell therapies face many challenges but hold great promise for improving clinical outcomes for patients with solid tumors. The field of ACT is growing exponentially.

## 6. Cancer Stem-Like Cell Therapy

CSCs (cancer stem-like cells) have been known to improve the efficacy of cancer therapy. Triptolide (C1572) selectively depleted CSCs in a dose-dependent manner in TNBC cell lines [127]. Nanomolar concentrations of C1572 markedly reduced c-MYC (MYC) protein levels via a proteasome-dependent mechanism [128,129]. Silencing MYC expression phenocopied the CSC-depletion effects of C1572 and induced senescence in TNBC cells [129]. Limited dilution assays revealed that ex vivo treatment of TNBC cells with C1572 reduced CSC levels by 28-fold [127]. In mouse xenograft models of human TNBC, administration of C1572 suppressed tumor growth and depleted CSCs in a manner correlated with diminished MYC expression in residual tumor tissues. Together, these findings provide a preclinical proof of concept defining C1572 as a promising therapeutic agent to eradicate CSCs for drug-resistant TNBC treatment [127,128,129]. Selinexor (KPT-330) is an oral SINE targeting Exportin 1 (XPO1). XPO1 functions as a nuclear exporter of major tumor suppressor proteins (TSPs) [130]. A phase II trial evaluated the safety, pharmacodynamics, and efficacy of selinexor (KPT-330), an oral selective inhibitor of nuclear export (SINE) in patients with TNBC [130]. Selinexor was well tolerated in patients with advanced TNBC but did not result in objective responses [127,128,129,130,131]. However, the clinical benefit rate was 30%, and further investigation of selinexor in this patient population should focus on combination therapies.

## 7. Future Prospective

TNBC is an aggressive malignancy associated with poor survival. So far, clinical trials have shown promising early-phase results. For instance, capecitabine has a significant response in patients with TNBC. Sacituzumab govitecan is likely to be approved for a phase II randomized trial and the ongoing ASCENT trial. Immunoconjugates such as sacituzumab govitecan have shown activity in patients with TNBC. The immunotherapeutic agent atezolizumab, with paclitaxel, is now approved for PD-L1-positive cancer patients. However, more immune-related drugs are required for chemotherapy and immune cell exhaustion. Tumor-specific target-based drugs can be developed against activators of tumor progression. Cell-based therapy can be used for tumor-specific mutations at specific targets. Thus, immunoconjugates are a crucial area of research for TNBC treatment. Finally, specific lesions, FGFR, AKT amplification, and specific mutations can also be targeted. There are ongoing trials for PI3 kinase and AKT inhibitors in TNBC, and whole genomic targeting is also being investigated.

## Figures and Tables

**Table 1:** Structures and efficacy of PARP inhibitors currently under clinical evaluation for TNBC.

Name	Mechanism	Clinical Efficacy	Type of Patent	Status	NCT Number
AZD2281, LYNPARZATM, and Ku-0059436	PARP1/2 inhibitor (Selective)	HER2-negative treated mTNBC, PARP Inhibitor, BRCA Mutated	Olaparib alone, with combination, durvalumab MEDI4736 against PD-L1	Phase I/II	NCT00679783 NCT03544125 NCT02484404 NCT03167619 NCT02681562 NCT02484404
		Inhibitor of Ataxia Telangiectasia and WEE1 inhibitor	Olaparib or olaparib in combination with AZD6738 Mutated (ATM) and AZD1775 in patent with TNBC	Phase II	NCT03330847
		Inhibitor of Ataxia Telangiectasia	Olaparib with radiation therapy, after chemotherapy in a patient with TNBC.	Phase I	NCT03109080
		Inhibitor of PD-L1	Olaparib with atezolizumab in TNBC	Phase II	NCT02849496
		Inhibitor of germline BRCA mutated	Olaparib with paclitaxel and carboplatin in TNBC	Phase II/III	NCT03150576, NCT02789332
		Inhibitor of VEGFR tyrosine kinase in recurrent TNBC	Olaparib with AZD2171 orally	Phase I/II	NCT01116648
		Inhibitor of BKM120	Olaparib with PI3K inhibitor, BKM120 in recurrent TNBC	Phase I	NCT01623349
		Inhibitor of heat shock protein 90 inhibitor	Olaparib with onalespib in TNBC	Phase I	NCT02898207
		mTORC1/2 inhibitor or Oral AKT inhibitor	Olaparib with AZD2014, in TNBC	Phase I/II	NCT02208375
Veliparib	PARP1/2 inhibitor	Inhibitor of EGFR and HER2, BRCA tyrosine kinase inhibitor	Veliparib in combination with cyclophosphamide	Phase II and failed in phases III trials	NCT01306032
			Veliparib alone	Completed phase I study of	NCT00892736
			Veliparib in combination with carboplatin	Completed phase I study	NCT01251874
			Veliparib with vinorelbine	Completed phase I	NCT01281150
			Veliparib with cisplatin	Completed phase I	NCT01104259
			Veliparib with pegylated	Completed phase I	NCT01145430
			Veliparib with lapatinib	Phase I	NCT02158507
			Veliparib in combined with irinotecan HCl	Phase I l	NCT00576654
			Veliparib with cisplatin	Phase II	NCT02595905
			Veliparib plus carboplatin	Phase III	NCT02032277
Iniparib	BSI-201 and SAR240550 (competitive PARP inhibitor)	Ability to form adducts with many cysteine-containing proteins	Combination with gemcitabine and carboplatin.	Phase II	NCT00813956 NCT01045304 NCT01130259
			Combination of iniparib with paclitaxel for TNBC compared to paclitaxel alone	Competed for phase II	NCT01204125
			Iniparib with irinotecan	Phase II trial of	NCT01173497

**Table 2:** Nanomedicine for triple-negative breast cancer theranostics.

Nanoparticle	Properties	Application	Status	Evidence	References
Quantum dots	High fluorescent intensity	Quantitative detection and Imaging in TNBC	Clinical ongoing	Applied in TNBCs patent for prognosis in immunohistochemistry (IHC) fluorescent signaling	[87]
Gold nano-stars	Increased optoelectronics	Therapy Photodynamic Drug delivery Hyperthermia	Experimental ongoing	sT1-signal for RMI imaging and photothermal therapy for TNBC	[88]
Nanocages	Used to deliver peptides, nucleic acids, and drugs	Hyperthermia Imaging Immunotherapy Photodynamics	Clinical ongoing	Used in therapy with gold nanocages on TNBC	[83]
Nanorods	Increased magnetic-optoelectronics capacity	Immunotherapy Photodynamics Hyperthermia Imaging Drug Delivery	Experimental/clinical ongoing	Deliver cisplatin therapy on TNBC	[88]
Nanocomposites	Increased nucleic acids, peptides, and drug-releasing with enhanced specificity.	Theranostics Gene Therapy Immunotherapy Photodynamic	Clinical ongoing	On using immunotherapy nanocomposites vehicle on TNBC	[89]
Nano-matryoshkas	Nanoparticles can deliver multiple drug payloads	Imaging and drug delivery	Clinical ongoing	Hyperthermia used in MDA-MB-231 murine xenograft study	[90]
Superparamagnetic iron oxide nanoparticles (SPIONs)	SPIONs have ability to spin alignment to an external magnetic field	SPIONs can apoptosis by using hyperthermia as well as real-time images of the tumors	Clinical ongoing	SPIONs are often used in TNBC MDA-MB-231 therapeutics	[91]
Fluorescent nano-diamonds (FNDs)	Tunable-enhanced optoelectronics	Enhance sensitivity and specificity Samarium-183 and Strontium-89, Iodine-131, Technetium-99 Nuclear medicine for enhancing	Clinical ongoing	Nanomaterials often used in MDA-MB-231 theranostics	[75,76]
Core-shell nanoparticles	Enhancing photodynamic to generate apoptosis for cancer theranostics	Enhanced frequencies to the magnetic field	Clinical Ongoing	SPION intravenously for cancer theranostics	[76,77]
AgNPs	Ag affects cellular microenvironment	Therapeutics by using cytotoxicity	Clinical ongoing	AgNP reduces TNBC growth in radiation therapy	[78]
IONP (Iron oxide nanoparticles)	Increased optoelectronics and magnetic features	Produce strong contrast images in MRI in T1 and T2	Clinical ongoing	MRI diagnostic on TNBC	[79]

**Table 3:** Clinical trials in the area of nanotechnology and TNBC.

Table 01525966.	Interventions with Drugs	Status	ClinicalTrials.gov Identifier
Trial of Neoadjuvant Chemotherapy +Carboplatin + NAB-Paclitaxel	Carboplatin, Paclitaxel Albuminstabilized nanoparticle laboratory biomarker analysis	Phase II Patients with Locally Advanced and Inflammatory TNBC	NCT01525966
A Randomized, Placebo-Controlled, Double-Blind of Nanoparticle Albumin-Bound Paclitaxel (Nab-Paclitaxel, Abraxane^®^) With or Without Mifepristone for Advanced, Glucocorticoid Receptor-Positive, TNBC	Drug: mifepristone Other: placebo Drug: nab paclitaxel	Phase II Trial	NCT02788981
Study of CORT125134 in Combination with Nab-paclitaxel in Patients with Solid Tumors	Drug: CORT125134 with nab paclitaxel	Phase 1/2	NCT02762981
Combined Targeted Therapies for Triple-Negative Advanced Breast Cancer—A of Weekly Nab-Paclitaxel and Bevacizumab Followed by Maintenance Targeted Therapy with Bevacizumab and Erlotinib	Drug: paclitaxel albumin stabilized nanoparticle formulation Biologic: bevacizumab Drug: erlotinib hydrochloride Other: laboratory biomarker analysis	Phase II Trial	NCT00733408
Efficacy and Tolerability of Nanoparticle Albumin-Bound Paclitaxel (Abraxane) in Patients with Metastatic Breast Cancer	Paclitaxel albumin stabilized nanoparticle formulation	phase -II	NCT01463072
Alone ABT-888 in Patients with Either BRCA 1/2 -Mutated Cancer	Veliparib	A Phase 1	NCT00892736
Pembrolizumab in Combination with Nab-paclitaxel Followed by Pembrolizumab in Combination with Cyclophosphamide and Epirubicin in Patients with TNBC	Drug: pembrolizumab Drug: nab paclitaxel Drug: epirubicin Drug: cyclophosphamide	Phase II	NCT03289819
Carboplatin, Abraxane, and Bevacizumab in mTNBC	Abraxane bevacizumab carboplatin	A Phase II	NCT00479674
AZD2281 (KU-0059436) Combined with Carboplatin in BRCA1/2 Mutation Carriers	Drug: AZ2281+carboplatin	Phase I	NCT01445418
Trabectedin in mTNBC patient with BRCA2 Mutation Carriers	Dexamethasone trabectedin	Phase II,	NCT00580112
Nab^®^-Paclitaxel with Gemcitabine or Carboplatin, as First-Line Treatment in TNBC	Abraxane Carboplatin	Phase 2/3,	NCT01881230

**Table 4:** Clinical trials of PD-L1 combination therapy with conventional cytotoxic chemotherapeutics targeting TNBC patients.

Drugs	Tested Patients	Combination with	Phase	Trial
Pembrolizumab	Neoadjuvant treatment for TNBC	Cyclophosphamide Paclitaxel Nab-paclitaxel Doxorubicin Carboplatin	I	NCT02622074;
Pembrolizumab	Metastatic TNBC (mTNBC)	Gemcitabine Carboplatin	II	NCT02755272
Pembrolizumab	Neoadjuvant and Adjuvant treatment for TNBC	Doxorubicin Epirubicin Cyclophosphamide Placebo Carboplatin Paclitaxel	III	NCT03036488
Pembrolizumab	Metastatic TNBC (mTNBC)	Capecitabine Paclitaxel	I/II	NCT02734290
Pembrolizumab	Metastatic TNBC (mTNBC)	Eribulin	Ib/II	NCT02513472;
Pembrolizumab	Metastatic TNBC (mTNBC)	Radiotherapy	II	NCT02730130
Pembrolizumab	Metastatic TNBC (mTNBC)	Cyclophosphamide	II	NCT02768701
Pembrolizumab	Metastatic TNBC (mTNBC)	Paclitaxel Gemcitabine Carboplatin Nab-paclitaxel	II	NCT02819518;
Durvalumab	Locally Advanced TNBC	Paclitaxe Epirubicin Cyclophosphamide	II	NCT03356860
Durvalumab		Nab-paclitaxel Epirubicin Cyclophosphamide	II	NCT02685059
Durvalumab	Clinical Stage I-III TNBC	Nab-paclitaxel Dose-dense doxorubicin/cyclophosphamide (ddAC)	I/II	NCT02489448
Durvalumab	mTNBC patients	Paclitaxel	I/II	NCT02628132
Durvalumab	First-line chemotherapy TNBC patients	Nab-paclitaxel + carboplatin + tremelimmab+Gemcitabine + carboplatin + tremelimumab	Ib	NCT02658214
Durvalumab	Metastatic TNBC (mTNBC)	Carboplatin Gemcitabine Hydrochloride Nab-paclitaxel Neoantigen vaccine	II	NCT03606967
Durvalumab	mTNBC patent	Carboplatin Paclitaxel Oleclumab (MEDI9447; anti-CD73)	I/II	NCT03616886
Atezolizumab	Advanced TNBC patient	Paclitaxel	Ib	NCT01633970
Atezolizumab	mTNBC patient	Nab-Paclitaxel Placebo	III	NCT02425891
Atezolizumab	Neoadjuvant treatment for TNBC	Anthracyclin, Abraxane Carboplatin, M PDL3280A	III	NCT02620280
Nivolumab	mTNBC patient	Cisplatin Romidepsin	I/II	NCT02393794

**Table 5:** Monotherapy and chemotherapy anti-PD-1/L1 trials in metastatic TNBC.

Regimen	Prior Lines	PD-L1	Number of Participants	ORR (Overall Response Rate), %	Median PFS (Progression-Free Survival) (95% CI), mo	Median OS (Overall Survival) (95% CI), mo	Trial/ClinicalTrials.gov Identifier
**Monotherapy trials**							
Pembrolizumab	44% ≥3 (min 1)	1 or –	170	5.3	2.0 (1.9–2.0)	9.0 (7.6–11.2)	KEYNOTE-086A NCT02447003
	40% ≥3 (min 1)	+ (CPS ≥1)	105	5.7	2.0 (1.9–2.1)	8.8 (7.1–11.2)	
	50% ≥3 (min 1)	–	64	4.7	1.9 (1.7–2.0)	9.7 (6.2–12.6)	
Pembrolizumab	Median: 2 (0–9)	1 (stroma≥% TC)	32	18.5	1.9 (1.7–5.5)		KEYNOTE-012 NCT01848834
Pembrolizumab	0	+ (CPS ≥1)	84	21.4	2.1 (2.0–2.2)	18.0 (12.9–23.0)	KEYNOTE-086B NCT02447003
Pembrolizumab vs. chemotherapy	1–2 (prior taxane 1) anthracycline	+ (CPS ≥1) or –	622	9.6 vs. 10.6	2.1 vs. 3.3; HR, 1.60 (1.33–1.92)	9.9 vs. 10.8; HR, 0.97 (0.82–1.15)	KEYNOTE-119 NCT02555657
		CPS ≥1	405	12.3 vs. 9.4	2.1 vs. 3.1; HR, 1.35 (1.08–1.68)	10.7 vs. 10.2; HR, 0.86 (0.69–1.06)	
		CPS ≥10	194	17.7 vs. 9.2	2.1 vs. 3.4; HR, 1.14 (0.82–1.59)	12.7 vs. 11.6; HR, 0.78 (0.57–1.06)	
		CPS ≥20	109	26.3 vs. 11.5	3.4 vs. 2.4; HR, 0.76 (0.49–1.18)	14.9 vs. 12.5; HR, 0.58 (0.38–0.88)	
Avelumab	Median: 2 (1–6)	+ or –	58	5.2	5.9 (5.7–6.9)	9.2 (4.3–NR)	JAVELIN NCT01772004
		+ (≥10 IC)	9	22.2			
		– (≥10 IC)	39	2.6			
Atezolizumab	58≥ 2	78% + (≥10 IC)	115	10	1.4 (1.3–1.6)	8.9 (7.0–12.6)	NCT01375842
**Chemotherapy combination trials**							
Pembrolizumab + eribulin	0–2	+ or –	106	26.4	4.2 (4.1–5.6)	17.7 (13.7–NR)	ENHANCE-1 NCT02513472
	0	+ or –	65	29.2	4.9 (4.1–6.1)	17.7 (13.3–NR)	
	1–2	+ or –	41	22.0	4.1 (2.1–6.2)	16.3 (12.4–19.2)	
Nab-paclitaxel + atezolizumab	0 (DFS ≥ 12 mo)	+ or –	902	56.0 vs. 45.9	7.2 vs. 5.5; HR, 0.80 (0.69–0.92)	21.0 vs. 18.7; HR, 0.85 (0.72–1.02)	IMpassion130 NCT02425891
	0 (DFS ≥ 12 mo)	+ (≥1% IC)	369	58.9 vs. 42.6	7.5 vs. 5.0; HR, 0.62 (0.49–0.78)	25.0 vs. 18.0; HR, 0.71 (0.54–0.93)	
Atezolizumab + nab-paclitaxel	0–2	+ or –	33	39.4	9.1 (2.0–20.9)	14.7 (10.1–NR)	NCT01375842

**Table 6:** Resulted targeted therapy and novel immunotherapy agent anti-PD-1/L1 trials in metastatic TNBC.

Regimen	Prior Line	Biomarker	Number of Participants	ORR (Overall Response Rate), %	Median PFS (Progression-Free Survival) (95% CI), mo	Median OS (Overall Survival) (95% CI), mo	Trial/ClinicalTrials.gov Identifier
**Monotherapy trials**							
Niraparib +pembrolizumab (PARP inhibitors)	1–3	PD-L1 + or –, BRCAm + or –	55	21	2.3 (2.1–3.9)		TOPACIO/ KEYNOTE-162 NCT02657889
		BRCAm + or –	15	47	8.3 (2.1–NR)		
		BRCAm –	27	11	2.1 (1.4–2.5)		
Nab-paclitaxel vs. paclitaxel + cobimetinib + atezolizumab (MEK inhibitors)	0	PD-L1 + or –	90	29.0 vs. 34.4	7.0 (3.7–12.8) vs. 3.8 (3.0–7.4)	NR (10.2–NR) vs. 11.0 (9.5–NR)	COLET NCT02322814
Nab-/paclitaxel + ipatasertib + atezolizumab (AKT inhibitors)	0 (DFS ≥12 mo)	PD-L1 + or –	26	73			Schmid AACR NCT03800836
Olaparib + durvalumab after 4 wk run-in (PARP inhibitors)	≤ 2	Germline BRCAm	17	58.8	4.9	20.5	MEDIOLA NCT02734004
Intratumoral c-MET mRNA CAR T cells (CAR T cells)	Any	PD-L1 + or –	6	0			NCT01837602
NKTR-214 + nivolumab (IL-2 agonists)	0–2	PD-L1 + or –	38	13.2			PIVOT-02 NCT02983045

**Table 7:** Ongoing anti-PD-1/L1 novel immunotherapy trials in TNBC.

Regimen	Line or Stage	Primary Endpoint	N	Trial/ClinicalTrials.gov Identifier
Chemotherapy (carboplatin + gemcitabine, or capecitabine) ± atezolizumab	0 (DFS ≥12 mo)	OS	350	IMpassion132 NCT03371017
Olaparib (PARPi) ± durvalumab: sporadic or germline BRCAm	≤ 2 including current platinum	PFS	60	DORA NCT03167619
Chemotherapy (carboplatin + gemcitabine, or nab-/paclitaxel) ± pembrolizumab	0 (DFS ≥6 mo)	PFS (Progression-free Survival), OS (overall survival)	847	KEYNOTE-355 NCT02819518
Paclitaxel ± atezolizumab	0	PFS	600	IMpassion131 NCT03125902
Olaparib (PARPi) ± atezolizumab: BRCAm (BRCA mutation)-positive	any	PFS	72	ETCTN NCT02849496
Paclitaxel ± ipatasertib (AKTi) ± atezolizumab	(DFS ≥12 mo)	PFS	450	IPATunity130 NCT03337724
Avelumab + binimetinib (MEKi) or utomilumab (IgG2 antibody) or anti-OX40 antibody	0–3	ORR	150	InCITe NCT03971409
Paclitaxel ± pembrolizumab X4 + SD-101 X 6 → AC x 4 → surgery	Stage II–III	Estimated pCR	TBD	I-SPY 2 NCT01042379
PVX-410 vaccine + pembrolizumab: HLAA2– positive	>1	Immune	20	NCT03362060
Paclitaxel 1 durvalumab ±capivasertib (AKTi) or danvatirsen (STAT3i) or oleclumab (anti-CD73)	0	AE (adverse event) rate	120	BEGONIA NCT03742102
Adjuvant neoantigen DNA vaccine ± durvalumab	RCB post NACT (neoadjuvant chemotherapy)	Safety	24	NCT03199040
Cyclophosphamide ±folate receptor a vaccine	≥T1c/≥N1/RCB (residual cancer burden)	DFS	280	NCT03012100
Adjuvant PVX-410 vaccine + durvalumab	Stage II–III	AE rate	22	NCT02826434
Adenovirus-mediated expression of HSV (Herpes simplex virus) thymidine kinase +valacyclovir + SBRT (stereotactic body radiation therapy) → pembrolizumab	>1	ORR	57	NCT03004183
Autologous TILs (LN-145) with intravenous IL-2	0–3	ORR, AEs (adverse event)	10	NCT04111510
Cyclophosphamide → mesothelin-targeted CAR T-cell	>1	MTD (Maximum Tolerated Dose)	36	NCT02792114
Gemcitabine 1 carboplatin 3 18 weeks → nab-paclitaxel + durvalumab ± Neoantigen vaccine	0	PFS	70	NCT03606967

## Data Availability

Data sharing not applicable.
